# “Cure” for multiple sclerosis (MS)—Evolving views of therapy goals in patients on different stages of the disease: A pilot study in a cohort of Polish MS patients

**DOI:** 10.1002/brb3.701

**Published:** 2017-05-02

**Authors:** Weronika Chacińska, Marta Brzostowska, Monika Nojszewska, Aleksandra Podlecka‐Piętowska, Wiesław W. Jędrzejczak, Emilian Snarski

**Affiliations:** ^1^ Department of Hematology Oncology and Internal DiseasesMedical University of WarsawWarsawPoland; ^2^ Department of NeurologyMedical University of WarsawWarsawPoland

**Keywords:** cure, mortality, multiple sclerosis, therapy goal, therapy risk

## Abstract

**Introduction:**

New aggressive treatments promise improvement of results in the treatment of multiple sclerosis (MS), however, with high risk of serious complications. In this study, we analyzed patients’ acceptance for risks connected with the MS treatment.

**Methods:**

The study was designed as a prospective nonanonymous online questionnaire. Responders were asked about the definition of the “cure” for MS and crucial goals in the treatment.

**Results:**

One hundred and eighty patients filled in the questionnaire (129 women and 51 men), and the mean age was 33 years (*SD* = 10.29). The MS forms were as follows: relapsing‐remitting (65%), secondary progressive (14%), primary progressive (10%), and other (11%), with mean EDSS score of 3 points (*SD* = 2.6). For 50% of the patients, relief of symptoms such as fatigue (72%), paresis (66%), and balance disorders (65%) was synonymous with “cure.” The patients with faster progression of the disease were likely to accept risky “curative” treatments—with average 68% accepted mortality risk (*p* = .003). Over 81% of patients accepted mortality rates over 1% for the treatment that achieves self‐defined cure.

**Conclusion:**

The study shows that the MS patients are likely to accept even very risky treatments as long as they promise patient‐defined “cure.”

## 1 Introduction

Multiple sclerosis (MS) is a demyelinating inflammatory disease of the central nervous system (CNS) and a leading cause of disability in young and middle‐aged people in the developed world. It affects over 2.3 million adults globally (Browne et al., 2014) The estimated prevalence of MS in 2013 was within a range from 0 to 5 per 100,000 to > 100 per 100,000 or even higher (about 200 per 100,000) in the newest studies from Norway and Canada (Browne et al., 2014; Grytten, Torkildsen, & Myhr, 2015; Kingwell et al., 2015). An increase in prevalence (predominantly due to longer survival) and incidence of MS over time in many places around the world with general increase in the incidence of MS in females has been observed in meta‐analyses of studies on MS epidemiology since 1965 (Koch‐Henriksen & Sorensen, 2010).

When making therapeutic decisions in MS it is important to know the potential risks and benefits of any individual therapy. Different therapeutic strategies are contemporary available for the treatment of relapsing‐remitting multiple sclerosis (RRMS). All of these medications have demonstrated partial efficacy along with different side effect profiles (Tramacere, Del Giovane, Salanti, D'Amico, & Filippini, 2015). Most patients with RRMS would start on a first‐line therapy but then be switched quickly to a second‐line medication with appearance of the disease activity (escalation therapy). This approach may lead in some patients to delay in initiation of more effective treatment. Alternatively, early treatment of RRMS cases with more potent immunosuppressive therapies with subsequent switch to weaker immunomodulatory agents (induction therapy) might translate into a greater efficacy, but potentially more serious side effects could be expected (Torkildsen, Myhr, & Bø, 2015; Vosoughi & Freedman, 2010). For a small group of patients who do not respond to the approved treatments, off‐label treatments like rituximab, ofatumumab, or experimental therapy with autologous haematopoietic stem cell transplantation (AHSCT) may be considered. These treatment have shown promising results in phase II and III trials or case series reports and seems to be effective against RRMS although not the progressive forms of the disease (Torkildsen et al., 2015).

New therapy options, especially natalizumab and alemtuzumab, are viewed as risky by neurologist and patients alike. This view is based on the most severe complications of those treatments: in postmarketing analyses from December 2015 overall incidence of progressive multifocal leukoencephalopathy (PML) is 4.11 per 1,000 natalizumab‐treated patients (with > 1% risk at 25–48 months of treatment in the highest risk group) (Biogen Medinfo). The overall rate of survival among patients with natalizumab‐associated PML is around 70%–80%, but patients who survive PML often experience serious morbidity, associated with substantial and permanent disability (McGuigan et al., 2016). Autoimmunity is the most important and substantial adverse event associated with alemtuzumab treatment. Among all, thyroid autoimmune disease, occurring in 30%–41% of patients, was the most common (Ruck, Bittner, Wiendl, & Meuth, 2015).

We have to keep in mind that drugs such as mitoxantrone, which is considered as dangerous by many, may lead to development of therapy‐related acute leukemia (TRAL). The overall risk of this complication is 0.73% (range: 0.25%–0.93% depending on country and treatment protocol, with number needed to harm 137.5 patients) compared with 0.003% for developing acute myelogenous leukemia (AML) in general population (Chan & Lo‐Coco, 2013; Ellis, Brown, & Boggild, 2015). Nearly 29% of TRAL patients died, which is similar to the mortality rate in spontaneously occurring AML. These data are comparable to the above‐mentioned high‐efficacy immunotherapies for MS.

Intense immunosuppression followed by AHSCT has been assessed as a possible new strategy in severe autoimmune disorders, particularly in highly active RRMS. Transplant‐related mortality of AHSCT, which was 5%–6% in the first reported series, has reduced since 2003 until now to 1%–2% (depending on the center and conditioning used) (Mancardi & Saccardi, 2008). This rapid reduction led to forming a proposition of a prospective, randomized, controlled multicenter clinical trial to assess the clinical efficacy of AHSCT for the treatment of aggressive MS (Saccardi et al., 2012).

The patients themselves in search of relief of symptoms are likely to follow different treatments—many times the hope for cure makes them seek alternatives that are rather unlikely to help them such as acupuncture or diet (National Multiple Sclerosis Society). The views of the patients on therapy risks change with the stage of the disease—the more desperate the patient the more likely he or she might look for the promise of cure.

So far there was no analysis of the patients evolving views on their treatment goals. What patients would consider as a “cure” for MS, what risks would they accept if such a “cure” would be achievable with given treatment options? With broader use of more risky treatments, we wanted to know what risks are acceptable by patients at different stages of the disease and how those views evolve during the disease. Moreover, with the introduction of the new treatment options, we wanted to see what percentage of patients is likely to accept treatment with higher mortality risk if this treatment promises “cure” within the patient's definition of what that entails.

## 2 Materials and Methods

The study was designed as a nonanonymous, voluntary, online questionnaire published on web forum “MS Fight for yourself” and “Neuropositive” for the patients who suffer from MS (Walcz o siebie; Neuropozytywni). The gathered data were analyzed anonymously by investigator. The protocol, which included patient information, formal patient consent for inclusion into the study and data analysis by investigator has been approved by the Bioethical Committee at the Medical University of Warsaw. The questionnaire consists of 34 items. Responders were asked about their basic personal data, details of their illness, the influence on personal life and professional qualifications, process of treatment, the patient's definition of the cure for MS and crucial goals in the treatment, but also the risk of death accepted by them if their therapy could lead to cure or satisfactory outcome at the current stage of the disease. Prior to being used, the questionnaire was validated with help of three MS patients and two neurologists (MN, APP). The final version of the file with questionnaire can be accessed online (Ankieta dla osoby chorej na stwardnienie rozsiane (SM)). The questionnaire was published between November 2014 and February 2015 on a social networking service to gain access to a possibly widest number of patients who suffer from MS.

### 2.1 Statistics

The OriginPro Student Version and Matlab R2015b Academic Licence (Academic Licence, producer The MathWorks, Inc. Protected by U. S and international patents) were used for statistical calculations (OriginPro Student Version). Differences between groups were analyzed with χ^*2*^ test using OriginPro Student Version. Statistical significance was set at *p* ≤ .05. The MATLABR2015b Academic Licence was used to calculate standard variation and arithmetic average.

## 3 Results

One hundred and eighty patients filled in the online questionnaire. There were no patients that were excluded from the study. Average age of the 176 patients was 33 years (range: 18 – 64 yrs; four patients did not report their age). The majority of the patients had Kurtzke Expanded Disability Status Scale (EDSS) score between 0 and 3 and were diagnosed with RRMS. The mean EDSS score was 3 points (*SD* = 2.6). The demographic data of the patients are presented in the Table 1.

**Table 1:** Demographic data of patients in the study (*n* = 180)

Sex	
Women	129 (72%)
Men	51 (28%)
Place of residence	
Countryside	20 (11%)
City	160 (89%)
City with population over 100 thousand	98 (54%)
Education	
Elementary education	4 (2%)
Vocational education	13 (7%)
Secondary education	57 (32%)
Higher education	100 (56%)
Other	6 (3%)
Current EDSS score	
0–3 points	101 (56.1%)
3.5–5 points	40 (22.2%)
5.5 points and more	33 (18.3%)
Did not report	6 (3.3%)
Type of MS	
Relapsing‐remitting MS	117 (65%)
Secondary‐progressive MS	26 (14%)
Primary‐progressive MS	18 (10%)
Another type of MS	8 (4%)
Do not know	11 (6%)

The patients were diagnosed with MS between 2 weeks and 22 years from the onset of the symptoms with 53.6% of the patients diagnosed within 1 year, 27.1% between 1 and 5 years, and for 15.4%, it took more than 5 years. Nearly 4% of the patients did not report the time from the first symptoms to the diagnosis.

**Figure 1:**
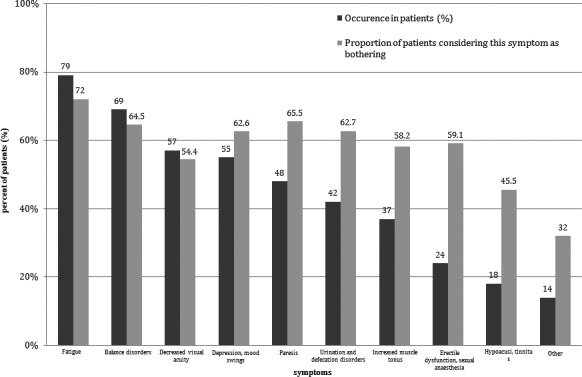
The occurence of MS symptoms reported by patients (*n* = 180)

Symptoms reported by more than 50% of the patients were fatigue (79%), balance disorders (69%), decreased visual acuity (57%), and depression/mood swings (55%). Of 10 of the most common symptoms, nine were considered as having a severe impact to their quality of life by more than 50% of the patients experiencing each of them. The detailed data on the symptoms of MS in the population studied are shown in Figure 1.

### 3.1 The patients’ definition of “cure”

We asked patients of what treatment goals would be synonymous with their definition of a “cure” for multiple sclerosis. Most of the patients (51%) pointed current symptoms relief as synonymous with “cure.” Other common answers were the removal of the cause of the disease (49%), full physical wellness (28%), stop of the disease progression at the current stage (25%), and mental wellness (22%).

**Figure 2:**
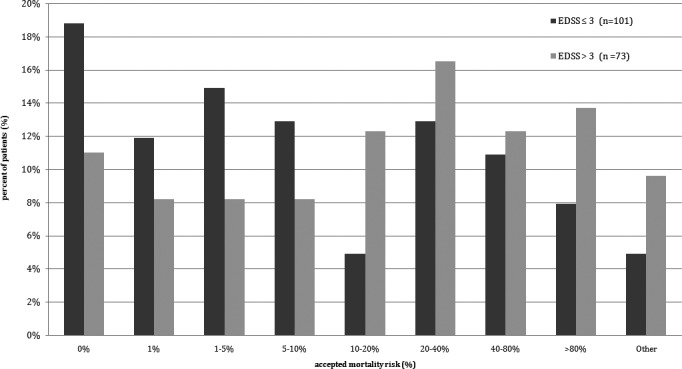
The mortality risk accepted by patients for the treatment that promises a “cure” for MS

To further investigate how important the self‐defined “cure” as the goal of the therapy was, we asked about accepted mortality risk if such goal was to be achieved. The treatment with mortality risk of 1% or higher was accepted by 81.2% patients with EDSS score between 0 and 3 points and 89.0% of the patients with EDSS score over 3 points. When the threshold level of mortality risk was set as 10% or higher, it was still accepted by 41.5% of the patients with lower EDSS score and 64.4% of the patients with higher EDSS score. Detailed data are presented in Figure 2.

As a “cure” is not possible in MS with current treatment options, we also asked the patients what would be satisfactory goal of the treatment at the current stage of the disease. As many as 62% of the patients regarded just stopping of the disease progression as satisfactory outcome. Other common answers included removal of the symptoms (45%), return to the clinical status prior to diagnosis (43%), and slowing the disease progression (12%).

**Figure 3:**
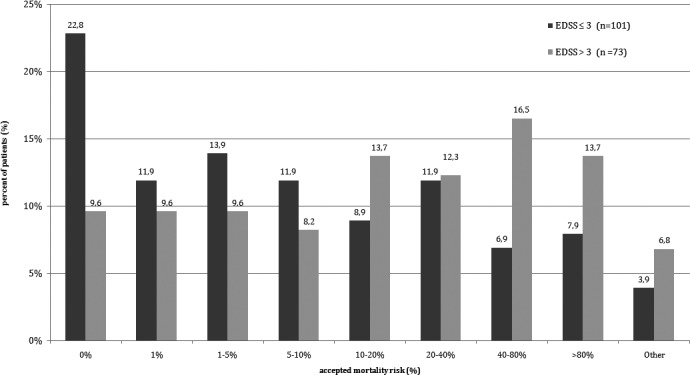
The mortality risk accepted by patients for the treatment offering a satisfactory goal at current stage of the disease

The patients were likely to undergo treatments with mortality risk as long as the therapy promised satisfactory goals for the patients. The treatment with mortality risk of 1% or higher was accepted by 57.5% patients with EDSS score between 0 and 3 points and by 90.4% of patients with more than 3 points in EDSS. When the mortality threshold of the treatment was set at 10%, there were still accepted by 39.5% of the patients with lower EDSS score and 63% of the patients with EDSS score over 3 points. Detailed data are shown in Figure 3.

**Figure 4:**
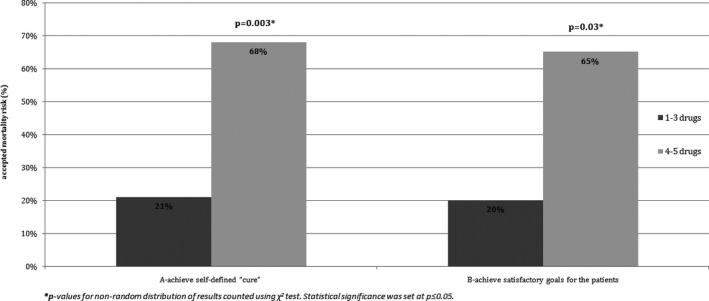
The correlation between the number of immunomodulatory drugs used in MS treatment and average accepted mortality risk for the treatment that A) achieve self‐defined “cure”; B) achieve satisfactory goals for the patients

The patients with more active disease, who undergo more treatment options in escalation algorithm or who started treatment with induction paradigm, were more likely to accept risky treatment as long as they promised self‐defined “cure” or achievement of satisfactory goals for the patients. The patients with history of use of more than three different drugs due to inadequate disease control were significantly more likely to accept more aggressive treatments than other patients if the therapy promised “cure” (*p* = .003) and also if the treatment promised other satisfactory goal (*p* = .03). Detailed data are shown in Figure 4.

The patients with history of using more than three different drugs had a mean EDSS score of 5 points (*SD* = 2.38) which differed significantly from average EDSS core of 3 points (*SD* = 2.53) in the studied population (*p* < .001).

When aims of the therapy were defined by the patients, 56% of them regarded stopping of the disease at the current stage with low‐risk therapy as the optimal treatment. The “cure” with high risk therapy was desired by 21% of the patients in the study. Partial relief of symptoms with medium‐risk therapy was satisfactory for 13% of the patients, while 10% of the patients gave answers that did not fit into those three approaches.

## 4 Discussion

What is the cure? The dictionary defines cure as something (such as a drug or medical treatment) that stops a disease and makes someone healthy again (Cure). When we look at the generally accepted treatments for MS, we cannot find any that could be termed “curative.” The MS patients still wait for such treatment that will bring not only “modification” or slowing the progression of the disease but would rather give results that could be termed “cure.” However, this definition of cure is a dictionary one. For many patients, “cure” is something different from such dictionary definition. It is true that the “cure” for MS within the patient definition is the removal of the cause of the disease. This is important for many of the patients and is a desired goal to the treating neurologists, but many of them are likely to accept somewhat smaller goals and term them as a “cure” for the disease—full physical wellness, relief of current symptoms, stop of the progression at the current stage of the disease, or mental wellness. So important indeed that the patients would regard them as a “cure” if they were to be achieved.

How important this self‐defined “cure” is? What risks vs benefit are the patients willing to accept to achieve it? We know that the most risky treatments of MS have mortalities within 1%. Results of our survey indicate that most of the patients are willing to accept such risks if the treatments promise patient‐defined “cure.” When the mortality threshold is set at 10%—still many of the patients are willing to undergo treatments as long as they can halt the progression of the disease. Actually patient self‐assessed accepted mortality threshold for treatment abolishing the symptoms of the disease is 20% for the patients with history of use of 1–3 different drugs for MS and 68% for the patients who tried more different drugs. This threshold is many times over any known experimental treatment of MS. The 68% threshold is higher than the 5‐year mortality rates connected with acute myeloid leukemia treated with allogenic bone marrow transplantation (Koenecke et al., 2015). This shows the desperation of the patients with their disease that does not respond to any treatment. Moreover, we have to be aware that patients, who are likely to accept treatments with such mortality rates, are quite likely to accept any treatment that promises “cure” and has no side effects—thus the “alternative” or stem cell therapies of MS have an easy auditorium among MS patients if they are not to be offered with evidence‐based alternatives.

The results of our study bring interesting insight into perceived risk of the treatment, but the study faces series of limitations. The patients assess the risk theoretically—they do not address the specific treatment risk before entering the treatment. This might actually explain relatively high accepted risks as there is a substantial difference between entering the study with 70% mortality or saying that such risk *would be* accepted. In our experience, experimental treatments with above zero mortality risk show that for many patients, lack of guarantee for 100% survival can be serious problem. On the other hand, the study addresses population of patients after many unsuccessful lines of therapy, without success in their treatment with progression of disability and without hope for any improvement—how would they behave if there was indeed a trial promising them “cure”? What is evident from the results is that there are some patients who accept the mortality risks well above the levels accepted by their physicians and similar to mortality in serious hematologic diseases. The group of patients in the study belongs to most engaged group of patients—they use computer as a mean of communication, are members of Internet patient forum, and actively participate in studies like ours—we are not sure whether the studied population is representative for the whole group of MS patients. The questionnaire assessed the basic disease data, but as they were patient reported and could not be verified by consulting physician, we are not fully able to be sure that the disease data for studied group are accurate and fully representative for the whole patient population. On the other hand, we designed nonanonymous questionnaire and only analyzed the results anonymously to improve quality of the data. Moreover, the study included different forms of MS and this may have influence on the number of possible therapy options offered to the patients.

Concluding, to our knowledge, it is the first report that addresses the questions of the optimal goals for MS therapy on different stages of the disease and mortality risk that the patients are ready to accept to achieve this goals. The perceived “cure” is defined rater by abolishing the symptoms than eradicating the disease. Mortality is important factor for patients that consider any treatment, but the perception of this risk can be easily overestimated or underestimated by physicians consulting patients depending on the attitude toward therapeutic option. Our report shows that the patients are desperate for treatments that do stop progression of multiple sclerosis, even if they are connected with significant mortality risk. This study was performed as a pilot study in a cohort of Polish MS patients, but we would like to extend its range on the territory of whole Europe to assess patients’ expectations considering the optimal goals and accepted risks for MS therapy.

## Conflict of Interest

The authors declare that there is no conflict of interest.
